# Health risks associated with social isolation in general and in young, middle and old age

**DOI:** 10.1371/journal.pone.0219663

**Published:** 2019-07-18

**Authors:** Oliver Hämmig

**Affiliations:** University of Zurich, Epidemiology, Biostatistics and Prevention Institute (EBPI), Zurich, Switzerland; Nathan S Kline Institute, UNITED STATES

## Abstract

**Introduction:**

Health effects of social isolation are well-studied at older age, in English-speaking countries, for individual health conditions, and based on unidimensional measures of isolation. Hardly any evidence exists for younger ages, for continental European and particularly German-speaking countries and based on multidimensional measures of isolation. This study therefore aimed to examine prevalence rates and associations of social isolation with various health conditions and behaviors in the entire Swiss population and across different age groups.

**Methods:**

Nationally representative cross-sectional data from the Swiss Health Survey collected in 2012 were used and analyzed. The study sample covered 21,597 randomly selected adolescents and adults living in Switzerland and aged 15 and older. A multifactorial five-item social integration index was used to assess social disconnectedness and perceived isolation.

**Results:**

Social isolation has been found to steadily increase with age and almost consistently to be strongly associated with poor health conditions and unfavorable behaviors across all ages. Nearly a quarter of the general population could be categorized as either only partly integrated or even poorly integrated and largely isolated. The socially isolated people independent of their age showed strongly elevated relative risks of poor self-rated health (aOR = 4.0), musculoskeletal disorders (aOR = 2.8), moderate to severe depression (aOR = 11.5), and multiple health problems (aOR = 5.0). They were also found to be at comparably high risk of behaving unhealthy with regard to physical inactivity (aOR = 2.2), poor diet (aOR = 1.9) and use of psychotropic medications (aOR = 3.6). Although prevalence rates of poor health conditions and behaviors differed greatly between the studied age groups, strong associations and clear dose-response relationships have been found separately for all age groups and particularly for the youngest. A fairly weak or no association at all (depending on the age group) with the degree of social integration was observed only for daily smoking.

**Conclusions:**

Social isolation may be less prevalent at younger ages, but is then even more strongly associated with poor health conditions and behaviors than at older ages.

## Introduction

There is an extraordinary ‘rise of living alone’ [1] in modern and ageing societies which is unique in social history and ‘among the most significant social changes of the modern world’ [2]. As early as 20 years ago, Killeen observed an ‘epidemic of loneliness’ [3]. One can argue about the importance of these phenomena, but there is a broad consensus that single-person households have been on the rise in recent decades and that ‘loneliness is far more prevalent in today’s society than it has been in previous generations’ [3]. And although living alone does not necessarily mean being isolated or feeling lonely, there is doubtless a certain connection between the two phenomena. Moreover, social isolation and loneliness are not only a social problem and of great relevance from a socio-historical point of view [1] but also a major health issue and a serious problem from a public health perspective [2].

Social isolation is understood here as the opposite of social integration or a lack of social interaction and therefore as having only few confidants or closely related persons or none at all. Loneliness as distinguished from isolation does not mean *being* alone and isolated, but *feeling* alone, unsupported and isolated (or socially disconnected). Social isolation and loneliness usually but not necessarily go along with each other, particularily not at younger and older ages. Young people not seldom feel lonely despite of being a full member in a group of peers or circle of friends. And elderly people not always feel lonely despite of a strongly reduced social network due to old age.

Social isolation, loneliness and hence a lack of social interaction and support have been a central concern of health research for several decades and are identified and well-documented risk factors for poor health (see i.a. [4–7]), unfavorable health behaviors [8, 9], increased morbidity [10–14] and early mortality [15–22]. But although social isolation is a widely known, increasingly recognized and broadly studied risk factor for morbidity and mortality, it is still not fully understood [9, 23–25]. While it is generally agreed that the negative health effects of social isolation are both direct and indirect (mediated by risky health behaviors), and although the research literature provides various behavioral, psychological and physiological pathways and mechanisms through which social isolation may influence or affect health [4, 14, 24, 26, 27], it remains unclear and/or has not been investigated as to which of them are most decisive–under what circumstances and in which populations and cultural settings.

Despite a variety of studies, the vast majority of research on social isolation or loneliness and health stems from the US or the UK. Recent studies from other European countries, and particularly from German-speaking regions, are largely lacking. Switzerland offers hardly any evidence on this issue with the exception of a single cross-sectional study that investigated the associations between social integration and support, including feelings of loneliness, on the one hand and depressive symptoms and disorders on the other [12].

Another shortcoming in the research literature is that the health risks associated with social isolation and/or loneliness have been mostly studied and found among the elderly. Thus, while the majority of studies have focused on social isolation in late adulthood or old age, only few looked at this issue in childhood, adolescence or early adulthood. In other words, there is extensive empirical evidence from older adults [6, 7, 9, 10, 21, 23], whereas evidence from youngsters [28], adolescents or young and middle-aged adults [29–31] is rather scarce. However, findings from longitudinal studies suggest that the health risks of social isolation represent long-term effects that have their origins much earlier in life [28, 32, 33].

In addition to these research gaps, a theoretical and methodological deficiency can also be observed in the literature. Definitions and conceptualizations of social isolation are often inconsistent and unidimensional [34, 35]. And although previous research has identified a wide range of indicators of social isolation, most studies look only at single or a few measures [7], often due to limited data [25]. They often focus either on objective, quantifiable aspects such as the number of social relationships or frequency of social contacts, or on subjective aspects such as the quality of social relationships or interactions [34]. Indicators of social isolation vary widely across studies and disciplines, and no broadly accepted concept or consolidated multiple-item measure for this complex, multidimensional construct has yet emerged and become established [23]. However, a number of indicators have been studied in relation to different health conditions [7]. Indicators and concepts of social isolation include a variety of elements such as living alone, being unmarried, having a small social network, participating infrequently in social activities, having few social contacts or feeling lonely and unsupported [7].

Some researchers distinguish between social isolation and loneliness, seeing them as two distinct concepts or phenomena that are only weakly connected with each other [36], while others do not make any conceptual distinction between the two categories at all or conceptualize social isolation and loneliness as just two different forms and/or measures of social isolation. According to the latter, loneliness is considered as the subjective perception of–or emotional response to–isolation, and equivalent to the objective lack of social integration and interaction [6, 7, 9]. According to Coyle and Dugan [6], loneliness is the distressing feeling of social isolation, whereby they assume a broad overlap or even complete congruence between these phenomena. However, it is undisputed that isolated people are not necessarily or always lonely, and lonely people are not necessarily isolated [36]. This is particularly the case as loneliness is considered to be a temporary state, even in later life [37]. However, it can be assumed that people who are socially isolated *and* feel lonely represent the tip of the iceberg of people with missing or deficient social relationships and contacts.

Against this background, the present study seeks to broadly examine social isolation and loneliness in association with health and health behaviors and to address all these shortcomings by using population-based and nationally representative Swiss data, by using an elaborate, self-constructed and well founded index of social integration as a two-dimensional measure of objective social isolation and subjective loneliness, by performing multiple-adjusted and age-stratified statistical analyses and by looking at diverse health conditions and behaviors.

This study addresses the following research questions:

- How prevalent is social isolation in the resident Swiss population in general and among different age groups in particular?
- Is social isolation consistently and equally associated with different health conditions and behaviors?
- Can similarly strong associations and clear dose-response relationships between social isolation and health (behaviors) be observed in all age groups?

## Methods

### Data and study sample

The data used for this study stem from the Swiss Health Survey 2012, a nationally representative sample survey among the permanent resident population in Switzerland aged 15 years and older living in private households. The survey is based on computer-assisted telephone interviews (CATI) followed by a written questionnaire, and provides broad and self-reported information on health status, health behavior and the use of health services on the one hand, and on a person’s predispositions and his/her natural, social and cultural environment on the other. The Swiss Health Survey is carried out every five years and the 2012 survey represents the fifth cross-sectional data collection since its launch in 1992.

Survey participants were selected by a random sampling of households and subsequently of people within these households, stratified by cantons and greater regions. The initial sample of the 2012 collection (the last wave currently available) consisted of 41,008 randomly selected persons aged 15 and older. Of these, 21,597 participated in the study and were questioned. Thus, the response rate was 52.7% and the sample was weighted and calibrated in order to guarantee its representative character and take account of the comparably large proportion of non-responders. The study took advantage of the full net sample taken. The study and the underlying statistical analyses were not subjected to any further restrictions.

No written consent of survey participants or formal approval by an ethics committee was needed as this study involved the use of a previously-published de-identified secondary data of the Swiss Health Survey. The data are collected on a voluntary basis, made anonymous for data users not allowing any conclusions to be drawn to individual persons, and used for statistical purposes only. The collection of self-reported health-related data by the Swiss Health Survey and within the resident population of Switzerland is officially approved and does not require further or formal approval by an ethical committee or authorisation by the commissioner for data protection nor are these recommended by the medical-ethical guidelines for scientific integrity of the Central Ethics Committee and the Swiss Academies of Sciences. Data protection and full anonymity is guaranteed by the Federal Statistics Act and the Data Protection Act and ensured by the data owner, the Swiss Federal Office of Statistics.

### Measures

#### Social integration or isolation

Swiss Health Survey data do not include an established and validated measure or scale of social integration or isolation. Therefore an own indicator had to be created based on available variables in the dataset. Following Cornwell and Waite [7], who suggested two forms or dimensions of social isolation, namely social disconnectedness and perceived isolation, an index consisting of five proxy variables that indicate these two dimensions more or less and, most importantly, that are included in the Swiss Health Survey and cover these two dimensions was constructed (see Table 1). According to Cornwell and Waite [7], a small social network with only few relationships, a lack of social interaction or contacts and a lack of participation in social activities are indicators of social disconnectedness, whereas the experience or perception of lack of social support and companionship as well as feelings of loneliness and not belonging indicate perceived isolation. The number of closely related persons to count on, the number of confidants among related persons, and the perceived concern and empathy of others in what one is doing were used as measures of *social disconnectedness*. Regretting the absence of a confidant or not, and the frequency of feelings of loneliness (from never to very often) were used as measures of *perceived isolation*. In this sense, social integration or isolation here is considered as the overarching theoretical construct which includes loneliness and not differentiates from loneliness.

**Table 1:** Forms and indicators of social integration or isolation selected from the Swiss Health Survey and results (factor loadings) of a principal component analysis.

Indicators (measures) by forms or dimensions of isolation	Components (factor loadings)	
	1	2
**Social disconnectedness** (objective dimension)		
**1. Low number of related persons to count on** ** **“How many people are so close to you that you can count on them in case of a serious personal problem?” (None / 1–2 persons / 3–5 persons / more than 5 persons)	**.77**	.09
**2. Low number of confidants among related persons** ** **“Is there someone with whom you really can talk about very personal problems among those persons who are related to you?” (Yes, several / yes, one single / no)	**.72**	.08
**3. Little concern by other people in what one is doing** ** **“How much interest and sympathy do other people show in and for what you’re doing?” (Very much / much / moderate / little / none	**.67**	.07
**Perceived isolation** (subjective dimension)		
**1. Regretting the absence of a confidant** ** **“Do you miss sometimes a person you really can talk with about personal problems at any time?” (Yes / no)	.06	**.81**
**2. Frequent feelings of loneliness** ** **“How often do you feel lonely?” (Very often / quite often / sometimes / never)	.11	**.79**

A factor or principal component analysis (with varimax rotation) of the five items or indicators produced a two-factor solution along the two conceptualized and theoretically identified dimensions or forms of isolation (suggested by Cornwell and Waite) and with an accumulated explained variance of 57% (Table 1). Nevertheless, an overall index with an aggregate score was calculated instead of two subscales for social disconnectedness and perceived isolation. Thus, all dichotomous or ordinally scaled variables were reclassified and recoded and weighted equally by assigning a score between 0 (no indication) and 2 (full indication) with a total score ranging from 0 to 10 indicating minimum to maximum integration (see Table 2). A reliability analysis of the five indicators or measures revealed a rather low Cronbach’s alpha of 0.53 as an estimate of internal consistency. This rather low alpha coefficient was to be expected in view of the two factors (with eigenvalues above 1) obtained by the principal component analysis. However, it demonstrates the still sufficient overall internal consistency of the two-dimensional multiple-item measure and above all justifies the construction and use of one single index instead of two subscales.

**Table 2:** Single indicators and overall index (sum score) of social integration or isolation, by sex and in total.

Indicators (variables)		Score	Men	Women	Total
**Number of related persons to count on in case of serious personal problems**					
	None to few (0–2 persons)	0	17.2%	17.8%	**17.5%**
	Several (3–5 persons)	1	44.1%	43.6%	**43.9%**
	Many (6 and more persons)	2	38.7%	38.6%	**38.6%**
**Number of confidants among related persons**					
	None (0)	0	4.4%	4.0%	**4.2%**
	One (1)	1	29.2%	24.5%	**26.8%**
	Multiple (2+)	2	66.4%	71.5%	**69.0%**
**Concern of other people in what one is doing**					
	None to little (1–2)	0	11.1%	10.0%	**10.5%**
	Moderate (3)	1	15.5%	11.9%	**13.7%**
	Much to very much (4–5)	2	73.3%	78.2%	**75.8%**
**Regretting the absence of a confidant**					
	Yes, sometimes	0	17.3%	25.0%	**21.2%**
	No	2	82.7%	75.0%	**78.8%**
**Feelings of loneliness**					
	Often to very often (1–2)	0	3.3%	5.4%	**4.4%**
	Sometimes (3)	1	26.4%	37.5%	**32.1%**
	Never (4)	2	70.3%	57.1%	**63.5%**
**Degree of social integration** (integration index)					
	Very low to low (isolated, not integrated at all)	0–4	6.6%	8.8%	**7.7%**
	Medium (partly integrated)	5–6	15.5%	16.2%	**15.8%**
	High (largely integrated)	7–8	34.9%	34.9%	**34.9%**
	Very high (fully integrated)	9–10	43.1%	40.1%	**41.6%**

Data source: **Swiss Health Survey 2012** (weighted data)

Strictly speaking, a reliability analysis is a reasonable test for a multi-item scale but is inappropriate for an index measuring a multi- or two-dimensional construct and latent variable such as social integration. An index is by definition not a scale with an internally consistent set of items which are designated as such a priori and therefore strongly interrelated and closely correlated with one another in measuring a unidimensional construct. Rather, it is a combination of different variables measuring different aspects or facets of an underlying multidimensional construct. In other words, having just few closely related persons to talk to if needed or only few persons to count on in case of serious problems, regretting the absence of a confidant from time to time or sometimes feeling lonely are, individually and alone, clearly insufficient measures of or proxies for social isolation and may only weakly correlate with each other [6, 7, 35, 38]. However, when they are combined and cumulated, they can be considered as indicative or predictive for “truly” being socially isolated.

#### Health conditions

Measures of four distinct aspects of health or disease were used for this study, namely self-rated health as an indicator of *general health*, combined (low) back pain and shoulder or neck complaints as a measure of *musculoskeletal health*, symptoms of depression as an indicator of *mental health* and an aggregate number of health problems as a proxy for *multimorbidity*.

To rate one’s own health as only moderate or even bad or very bad was categorized as poor self-rated health. Combined self-reports of suffering from (low) back pain and neck or shoulder pain with reported *strong* pain for both or at least one of these complaints were classified as musculoskeletal disorders. Depression was measured by using the nine-item Patient Health Questionnaire (PHG-9), a diagnostic instrument for mental disorders, with response categories on a four-point frequency scale from “not at all” (0) to “almost every day” (3). A sum score of 10 and more on a scale from 0 to 27 was considered as moderate (10–14) or even rather severe (15–19) and severe depression (20–27).

A cumulative number of three or more out of ten severe general, physical and mental health problems or poor health conditions was used as a measure of multimorbidity. These health problems cover self-reports of bad or very bad self-rated health (3.0% of the entire survey sample and study population), a chronic health problem (31.2%) and being strongly handicapped in daily life for health reasons (4.1%). Furthermore, suffering from strong (low) back pain (6.9%), strong neck or shoulder pain (6.9%), strong stomach pain (2.6%), strong headaches (5.0%), strong tachycardia or palpitations (1.0%), strong chest pain (0.9%), and from moderate to severe depression (6.5%) were additionally included in this index measuring multiple health problems or “multimorbidity”.

#### Health behaviors

Unfavorable behaviors used in this study were physical inactivity, an unhealthy diet, daily smoking and use of psychotropic medication. Physical activity was assessed by an index on the scale and intensity of weekly physical leisure activities. Respondents with less than 30 minutes of moderate physical activity and less than one period of intense physical activity per week were categorized as “inactive”. Combined fruit and vegetable under-consumption measured by less than five days per week of eating fruit (or drinking fruit juices) and/or vegetables or salads, was categorized as an “unhealthy diet”. Daily smoking must be distinguished from occasional smoking and was directly addressed (“Do you smoke–even if rarely?”, and if the answer is ‘Yes’: “Do you smoke daily?”). About three quarters of the smokers in the study population smoke daily and one quarter are occasional smokers. The use of psychotropic medications was assessed with the self-reported consumption of antidepressants and/or sedatives (tranquilizers) and/or sleeping pills during the past week.

### Analyses

All statistical analyses were based on weighted and extrapolated data in order to better represent the total population, to better estimate prevalence rates and to provide more statistical power by relating the projected number of cases to the whole population. Descriptive statistics were calculated to characterize the study sample as a whole and were differentiated by subgroups (sexes, age groups) and to estimate the proportion of socially integrated and isolated persons in the Swiss resident population aged 15 and older. In order to answer the research questions and to test the general validity, sensitivity and stability or consistency of the main finding with regard to the studied association between social isolation and health, further statistical analyses were performed for various health conditions and behaviors, using differently specified models and related to different population strata. Cross-tabulations as well as bi- and multivariate logistic regression analyses were carried out for eight health-related parameters separately to calculate the prevalence rates or relative frequencies (percentages) and relative risks (odds ratios) of poor health conditions and behaviors linked to different degrees of social integration. The associations were analyzed, first in unadjusted form and then adjusted in steps for the control variables and additional covariates. The fully adjusted and specified models for all considered health conditions and behaviors were then calculated separately for several age groups (15–24, 25–44, 45–64, 65+).

## Results

### Descriptive analyses

For further analyses, and in order to calculate the relative health (behavior) risks of the less integrated or isolated groups in comparison with the highly integrated ones, the self-constructed index of social integration as a measure of social disconnectedness and perceived isolation with a score ranging between 0 and 10 was categorized into four degrees of social integration (very low/low, medium, high, very high). This classification still and largely reflects the marginal distribution of the index which is fairly “normal”, i.e. strongly skewed to the left, but still bell-shaped and unimodal (not shown).

According to this categorization with cut-offs along the largely normal distribution of the index, nearly 8% of the study sample and therefore resident Swiss population aged 15 years and older are comparatively isolated and not integrated at all, and another 16% are only partly integrated (see Table 2). On the other hand, more than three quarters of the population are largely to fully integrated. A significant difference between the two sexes was found in this regard. As shown in Table 2, women aged 15 years and older much more often miss a confident and slightly more often feel lonely–or at least report feelings of loneliness–than men of the same age. Consequently, women seem to be socially isolated or only partly integrated to a slightly greater extent (25%) than men (22%).

Survey participants of older ages can be characterized in general by a higher proportion of native Swiss, less educated people, a greater number of people who are separated, divorced or widowed and who are living alone, a higher frequency of overweight and obese people and a higher proportion of persons with poor self-rated health (see Table 3). These characteristics, with the exception of household size, education and nationality, are true age effects which steadily and unsurprisingly become more frequent with age. In contrast, nationality and educational level are linked to a specific birth cohort.

**Table 3:** Descriptive statistics and characteristics of the study sample by age groups.

		Youngsters, adolescents (15–24 yrs)	Young and middle-aged adults (25–44 yrs)	Adults of advanced working age (45–64 yrs)	Elders / older adults (65+ yrs)	Total (15+ yrs)
**N**		2,350	5,252	6,628	4,127	**18,357**
**Age**, M (SD)		19.8 (2.7)	34.9 (5.9)	53.4 (5.8)	74.3 (6.8)	**47.4 (18.7)**
**Sex**						
	Men	51.1%	50.1%	50.6%	43.6%	**49.0%**
	Women	48.9%	49.9%	49.4%	56.4%	**51.0%**
**Marital status**						
	Single	97.1%	42.7%	10.9%	6.3%	**31.5%**
	Married / cohabiting	2.7%	50.2%	68.1%	58.3%	**51.7%**
	Divorced / separated / widowed	0.1%	7.1%	21.0%	35.4%	**16.8%**
**Size of household** (number of household members)						
	Single-person household (1)	2.0%	10.2%	10.8%	30.8%	**13.7%**
	Two-person household (2)	9.5%	25.4%	34.6%	63.0%	**34.1%**
	Multi-person household (3+)	88.5%	64.3%	54.6%	7.5%	**52.2%**
**Education** (highest level achieved)						
	Compulsory education only	7.1%	0.9%	2.1%	3.3%	**2.6%**
	0–2 years post-compulsory education	41.9%	12.0%	17.5%	29.8%	**21.5%**
	Basic vocational education	26.5%	39.9%	41.8%	43.1%	**39.5%**
	Higher vocational education	21.1%	28.3%	23.8%	15.1%	**23.1%**
	University degree	3.3%	18.9%	14.8%	8.7%	**13.3%**
**Nationality**						
	Native Swiss	70.6%	55.6%	66.5%	78.2%	**66.0%**
	Naturalized Swiss	9.2%	9.9%	12.5%	11.5%	**11.0%**
	No Swiss	20.2%	34.5%	21.0%	10.3%	**23.0%**
**Weight** (relative to body size)						
	Underweight (BMI <18)	7.3%	3.6%	2.2%	2.7%	**3.4%**
	Normal weight (BMI 18–25)	74.2%	62.4%	49.7%	44.2%	**55.8%**
	Overweight (BMI 25–30)	14.8%	26.7%	35.5%	38.9%	**30.7%**
	Obesity (BMI 30+)	3.6%	7.3%	12.6%	14.2%	**10.1%**
**Health status** (self-rated general health)						
	Very good	52.5%	49.4%	35.7%	22.2%	**39.5%**
	Good	41.2%	41.8%	45.6%	47.4%	**44.2%**
	Moderate	5.9%	7.3%	14.0%	25.7%	**13.3%**
	Bad	0.4%	1.1%	3.9%	4.1%	**2.6%**
	Very bad	0.1%	0.3%	0.8%	0.6%	**0.5%**
**Feelings of loneliness**						
	Often to very often (1–2)	3.6%	4.3%	4.6%	4.7%	**4.4%**
	Sometimes (3)	40.7%	35.1%	29.2%	26.7%	**32.1%**
	Never (4)	55.7%	60.6%	66.2%	68.6%	**63.5%**
**Degree of social integration** (integration index)						
	Very low to low (isolated)	3.5%	6.5%	7.9%	11.9%	**7.7%**
	Medium (partly integrated)	13.1%	13.6%	14.9%	22.7%	**15.8%**
	High (largely integrated)	36.3%	35.1%	34.6%	34.1%	**34.9%**
	Very high (fully integrated)	47.1%	44.8%	42.5%	31.4%	**41.6%**

Data source: **Swiss Health Survey 2012** (weighted data)

Loneliness and social isolation seem to be depending on age. Although frequent feelings of loneliness only slightly increase with age, social isolation increases substantially with age (see Table 3). The proportion of comparatively socially isolated persons rises steadily and gradually from below 4% among the youngsters to 12% among the elderly (see Table 3). And while one sixth of the youngest age group is socially isolated or only partly integrated, more than one third of the oldest age group can equally be classified.

### Bi- and multivariate association analyses

Social isolation is a matter of age as expected, but independent of age isolation is strongly associated with poor health (see Table 4) and unfavorable health behaviors (see Table 5). A clear gradient or dose-response relationship can also be observed in this association: the lower the degree of social integration, the higher the prevalence (percentage) and the relative risk (odds ratio) of being in poor self-rated health and having accumulated musculoskeletal disorders, depressive symptoms or several co-morbidities or health problems (see Table 4). The prevalence rates of poor health conditions among socially isolated survey participants were found to be more than two or three times higher than average and up to seven times higher than among the fully integrated group. While only 9% of the fully integrated individuals reported poor general health, 37% of the isolated ones rated their own health as only moderate to very bad. Around 3% to 5% of the fully integrated group but between 16% and 23% of the isolated group showed accumulated musculoskeletal disorders, depressive symptoms or multiple co-morbidities. Even when adjusted for control variables and covariates, the socially isolated or least integrated respondents showed a threefold and up to twelve-fold higher risk of being in poor general, musculoskeletal and mental health and having multiple health problems or co-morbidities compared to fully integrated participants.

**Table 4:** Prevalence rates and relative risks of poor health conditions, by degrees of social integration.

	Poor self-rated health (3–5)			Accumulated musculoskeletal disorders (3–4)			Moderate to severe depression (10–27)			Multimorbidity (3+ health problems)		
	%^1)^	OR	95% CI	%^1)^	OR	95% CI	%^1)^	OR	95% CI	%^1)^	OR	95% CI
Total study population	15.9			7.4			6.5			5.7		
**Model 1**^2)^												
**Degree of social integration**												
Very low to low (0–4)	37.4	**5.85**	5.81–5.89	15.5	**3.62**	3.58–3.65	23.3	**9.36**	9.23–9.45	16.3	**5.88**	5.82–5.94
Medium (5–6)	24.0	**3.10**	3.08–3.12	11.3	**2.50**	2.48–2.52	11.1	**3.84**	3.81–3.88	9.3	**3.07**	3.04–3.10
High (7–8)	15.3	**1.78**	1.77–1.79	6.9	**1.46**	1.45–1.47	5.4	**1.77**	1.76–1.79	5.1	**1.62**	1.60–1.63
Very high (9–10)	9.3	1		4.8	1		3.1	1		3.2	1	
No. of cases in model			17,832			17,837			16,587			16,522
**Model 2**^3)^												
**Degree of social integration**												
Very low to low (0–4)	37.4	**4.33**	4.30–4.36	15.5	**2.92**	2.89–2.95	23.3	**11.82**	11.7–11.9	16.3	**5.07**	5.01–5.12
Medium (5–6)	24.0	**2.37**	2.35–2.38	11.3	**2.17**	2.15–2.19	11.1	**4.27**	4.23–4.31	9.3	**2.79**	2.76–2.82
High (7–8)	15.3	**1.62**	1.61–1.63	6.9	**1.37**	1.36–1.38	5.4	**1.82**	1.80–1.83	5.1	**1.54**	1.52–1.55
Very high (9–10)	9.3	1		4.8	1		3.1	1		3.2	1	
No. of cases in model			17,798			17,803			16,558			16,493
**Model 3**^4)^												
**Degree of social integration**												
Very low to low (0–4)	37.4	**4.02**	3.99–4.05	15.5	**2.80**	2.77–2.82	23.3	**11.52**	11.4–11.6	16.3	**5.02**	4.97–5.08
Medium (5–6)	24.0	**2.31**	2.30–2.33	11.3	**2.14**	2.12–2.15	11.1	**4.25**	4.21–4.29	9.3	**2.81**	2.78–2.83
High (7–8)	15.3	**1.60**	1.59–1.61	6.9	**1.36**	1.35–1.37	5.4	**1.81**	1.79–1.83	5.1	**1.54**	1.53–1.56
Very high (9–10)	9.3	1		4.8	1		3.1			3.2	1	
No. of cases in model			17,689			17,692			16,466			16,403

Data source: **Swiss Health Survey 2012**

1) Prevalence rates (relative frequencies) according to weighted data

2) Unadjusted model: OR not adjusted for any control variables or covariates

3) Partly adjusted model: OR adjusted for sex, age and education

4) Fully adjusted model: OR additionally adjusted for foreign nationality and overweight/obesity (BMI >25)

**Table 5:** Prevalence rates and relative risks of health behaviors, by degrees of social integration.

	Physical inactivity			Unhealthy diet			Daily smoking			Use of psychotropic medication (last 7 days)		
	%^1)^	OR	95% CI	%^1)^	OR	95% CI	%^1)^	OR	95% CI	%^1)^	OR	95% CI
Total study population	10.1			9.7			20.3			19.1		
**Model 1**^2)^												
**Degree of social integration**												
Very low to low (0–4)	20.1	**3.55**	3.52–3.58	12.4	**1.59**	1.58–1.61	22.8	**1.25**	1.24–1.26	36.8	**4.12**	4.08–4.15
Medium (5–6)	15.1	**2.51**	2.49–2.52	12.5	**1.61**	1.60–1.63	21.6	**1.16**	1.15–1.16	23.9	**2.21**	2.20–2.23
High (7–8)	9.8	**1.53**	1.52–1.54	9.6	**1.20**	1.19–1.21	20.5	**1.09**	1.08–1.09	18.5	**1.59**	1.59–1.62
Very high (9–10)	6.6	1		8.1	1		19.2	1		12.4	1	
No. of cases in model			17,667			17,780			17,844			8,567
**Model 2**^3)^												
**Degree of social integration**												
Very low to low (0–4)	20.1	**2.62**	2.59–2.64	12.4	**1.98**	1.96–2.00	22.8	**1.36**	1.35–1.37	36.8	**3.59**	3.55–3.62
Medium (5–6)	15.1	**1.98**	1.97–2.00	12.5	**1.78**	1.77–1.79	21.6	**1.19**	1.18–1.19	23.9	**2.00**	1.98–2.02
High (7–8)	9.8	**1.40**	1.39–1.41	9.6	**1.24**	1.23–1.25	20.5	**1.09**	1.09–1.09	18.5	**1.54**	1.53–1.55
Very high (9–10)	6.6	1		8.1	1		19.2	1		12.4	1	
No. of cases in model			17,634			17,746			17,810			8,551
**Model 3**^4)^												
**Degree of social integration**												
Very low to low (0–4)	20.1	**2.18**	2.17–2.20	12.4	**1.91**	1.89–1.93	22.8	**1.26**	1.25–1.27	36.8	**3.64**	3.61–3.68
Medium (5–6)	15.1	**1.77**	1.76–1.78	12.5	**1.75**	1.74–1.77	21.6	**1.12**	1.12–1.13	23.9	**2.02**	2.01–2.04
High (7–8)	9.8	**1.28**	1.27–1.29	9.6	**1.24**	1.23–1.24	20.5	**1.06**	1.05–1.06	18.5	**1.55**	1.54–1.56
Very high (9–10)	6.6	1		8.1	1		19.2			12.4	1	
No. of cases in model			17,525			17,637			17,699			8,498

Data source: **Swiss Health Survey 2012**

^1)^ Prevalence rates (relative frequencies) according to weighted data

^2)^ Unadjusted model: OR not adjusted for any control variables or covariates

^3)^ Partly adjusted model: OR adjusted for sex, age and education

^4)^ Fully adjusted model: OR additionally adjusted for foreign nationality and overweight/obesity (BMI >25)

The associations between degrees of social integration and adverse health behaviors are much weaker, but still fairly strong and largely linear, with the exception of daily smoking (see Table 5). These associations do not become substantially weaker with a stepwise adjustment for control variables and additional covariates, if at all, except for physical inactivity. Multiple adjusted odds ratios for socially isolated compared to fully integrated participants range from 1.3 to 3.6 for unfavorable health behaviors such as physical inactivity, an unhealthy diet, daily smoking and use of psychotropic medications. The strongest associations and steepest gradients, i.e. the highest increase of prevalence rates and odds ratios (relative risks) were found for the use of psychotropic or psychoactive medications, and the weakest ones, i.e. the lowest increase of prevalence rates and odds ratios (relative risks) for daily smoking. While 12% of fully integrated Swiss residents regularly consume psychotropic medications, 37% of the comparably isolated group do so. And while only small proportions of the fully integrated group show unhealthy behaviors with regard to physical activity (7%) and diet (8%), at least every eighth member of the isolated group has a poor diet and every fifth member is physically inactive. Daily smoking is least prevalent among the fully integrated group (19%) and is most widespread among the socially isolated group (23%).

These gradients or dose-response relationships are even more pronounced in multivariate analyses after adjustment for different control variables and covariates. The odds ratio as a proxy for the relative risk consistently increases gradually and significantly with decreasing social integration.

#### Age-stratified analyses

Separate analyses for different age groups showed significantly different prevalence rates of poor health conditions on average and by degree of social integration between the four age groups but consistently strong associations and clear dose-response relationships throughout all age groups (see Table 6).

1. The group of youngsters and adolescents (age group 1) in total has the lowest prevalence rates of poor self-rated health (6%), accumulated musculoskeletal disorders (5%) and multiple health problems (4%), and the highest rate of moderate or major depression (11%). Particularly those young people with a (very) low degree of social integration show an extraordinary high prevalence rate of depressive disorders, at 48% the highest rate of all age groups and across all health conditions and degrees of social integration.
2. In the group of young and middle-aged adults (age group 2), prevalence rates are mostly higher (except for depression) and associations and dose-response relationships are mostly weaker compared to age group 1. However, the socially isolated members of this group still have a relatively high risk of poor health conditions. Prevalence rates vary between 13% and 30% and are thus far above average (6% to 9%), and multiple adjusted odds ratios range between 3.2 and 10.0.
3. Adults of advanced (working) age from age group 3 once more have substantially higher prevalence rates of poor health conditions than members of age group 2, with the exception of depression, which is significantly less prevalent among them. In this group, associations between the degree of social integration and poor health conditions are strongest and gradients most pronounced for depression and multimorbidity. Multiple adjusted odds ratios are similarily high than in age group 3 and vary from 4.5 to 14.1 for socially isolated members compared to the reference group of fully integrated persons.
4. Among elders and older adults (age group 4), prevalence rates are clearly highest for poor self-rated general health (30%) and lowest for poor mental health or depression (3%) of all age groups. The adjusted odds ratios as measures of associations and proxies for relative health risks for the most exposed (socially isolated) range between 2.5 and 18.1 und are almost consistently smaller than in age group 3.

**Table 6:** Prevalence rates and relative risks of poor health conditions, by degrees of social integration and by age.

	Poor self-rated health (3–5)			Accumulated musculoskeletal disorders (3–4)			Moderate to severe depression (10–27)			Multimorbidity (3+ health problems)		
	%^1)^	OR^2)^	95% CI	%^1)^	OR^2)^	95% CI	%^1)^	OR^2)^	95% CI	%^1)^	OR^2)^	95% CI
Total study population	16.3			7.5			6.5			5.7		
**Age group 1** (15–24 years)	**6.3**			**4.5**			**10.5**			**3.1**		
**Degree of social integration**												
Very low to low (0–4)	**18.1**	**4.59**	4.44–4.74	**11.5**	**4.01**	3.86–4.18	**47.9**	**16.14**	15.7–16.6	**13.9**	**6.98**	6.70–7.26
Medium (5–6)	**9.7**	**2.13**	2.08–2.18	**7.0**	**2.19**	2.12–2.25	**19.0**	**3.78**	3.70–3.86	**4.8**	**1.93**	1.86–2.00
High (7–8)	**6.3**	**1.34**	1.32–1.37	**5.0**	**1.51**	1.48–1.55	**10.9**	**2.03**	2.00–2.07	**2.8**	**1.19**	1.15–1.22
Very high (9–10)	**4.5**	1		**3.0**	1		**5.1**	1		**2.1**	1	
No. of cases in model			2,311			2,311			2,261			2,259
**Age group 2** (25–44 years)	**8.7**			**5.7**			**7.7**			**4.5**		
**Degree of social integration**												
Very low to low (0–4)	**27.2**	**5.80**	5.71–5.89	**13.1**	**3.16**	3.10–3.23	**30.3**	**9.98**	9.82–10.1	**14.5**	**6.50**	6.36–6.64
Medium (5–6)	**13.2**	**2.51**	2.48–2.55	**10.2**	**2.53**	2.49–2.57	**14.6**	**4.05**	3.99–4.11	**9.1**	**3.95**	3.88–4.02
High (7–8)	**8.0**	**1.48**	1.47–1.50	**4.8**	**1.14**	1.13–1.16	**5.9**	**1.48**	1.46–1.50	**3.6**	**1.49**	1.47–1.52
Very high (9–10)	**5.2**	1		**4.0**	1		**4.0**	1		**2.5**	1	
No. of cases in model			5,144			5,144			5,009			4,990
**Age group 3** (45–64 years)	**18.1**			**9.0**			**5.6**			**7.5**		
**Degree of social integration**												
Very low to low (0–4)	**40.0**	**4.56**	4.51–4.62	**18.7**	**3.11**	3.07–3.16	**21.5**	**11.14**	10.9–11.4	**20.2**	**5.93**	5.83–6.03
Medium (5–6)	**27.6**	**2.55**	2.52–2.58	**14.0**	**2.25**	2.22–2.28	**10.0**	**4.53**	4.44–4.61	**12.5**	**3.29**	3.24–3.34
High (7–8)	**17.9**	**1.72**	1.70–1.73	**8.4**	**1.42**	1.40–1.43	**4.7**	**2.07**	2.03–2.11	**7.3**	**1.91**	1.88–1.93
Very high (9–10)	**10.8**	1		**6.0**	1		**2.4**	1		**4.0**	1	
No. of cases in model			6,388			6,392			6,037			6,013
**Age group 4** (65+ years)	**30.0**			**9.3**			**2.9**			**6.6**		
**Degree of social integration**												
Very low to low (0–4)	**47.2**	**3.25**	3.21–3.30	**15.1**	**2.48**	2.44–2.53	**11.7**	**18.07**	17.3–18.9	**14.1**	**3.03**	2.96–3.10
Medium (5–6)	**36.0**	**2.26**	2.24–2.28	**11.2**	**1.97**	1.93–2.00	**4.0**	**6.20**	5.93–6.48	**7.7**	**1.69**	1.65–1.73
High (7–8)	**29.6**	**1.71**	1.69–1.72	**9.3**	**1.61**	1.58–1.63	**1.6**	**2.36**	2.25–2.47	**5.7**	**1.18**	1.16–1.21
Very high (9–10)	**19.7**	1		**5.9**	1		**0.7**	1		**4.6**	1	
No. of cases in model			3,846			3,845			3,159			3,141

Data source: **Swiss Health Survey 2012**

^1)^ Prevalence rates (relative frequencies) according to weighted data

^2)^ Fully adjusted model: OR adjusted for sex, education, foreign nationality and overweight/obesity (BMI >25)

As regards the studied health behaviors (see Table 7), the patterns are similar, but the associations and gradients are much less strong and not consistently linear with respect to the degree of social integration. The strongest associations with regard to these health behaviors and the highest prevalence rates and relative risks among the socially isolated were found for the use of psychotropic medications in young and middle age (least compared to most integrated: 31% vs. 9%, aOR = 4.3) und in advanced age (43% vs. 14%, aOR = 4.7), and in the elderly (38% vs. 16%, aOR = 3.0), and for physical inactivity (12% vs. 4%, aOR = 2.9) and unhealthy diet (35% vs. 13%, aOR = 3.5) in youth and adolescence. Rather weak or no (linear) associations at all between the degree of social integration and the corresponding health behaviors were found for daily smoking in all age groups.

**Table 7:** Prevalence rates and relative risks of unhealthy behaviors, by degrees of social integration and by age.

	Physical inactivity			Unhealthy diet			Daily smoking			Use of psychotropic medication (last 7 days)		
	%^1)^	OR^2)^	95% CI	%^1)^	OR^2)^	95% CI	%^1)^	OR^2)^	95% CI	%^1)^	OR^2)^	95% CI
Total study population	10.6			9.7			20.2			19.3		
**Age group 1** (15–24 years)	**5.4**			**14.8**			**22.4**			**9.3**		
**Degree of social integration**												
Very low to low (0–4)	**11.8**	**2.91**	2.80–3.03	**34.8**	**3.48**	3.39–3.57	**27.5**	**1.43**	1.39–1.46	**12.7**	**1.98**	1.86–2.10
Medium (5–6)	**10.7**	**2.59**	2.53–2.66	**18.3**	**1.45**	1.43–1.48	**24.8**	**1.31**	1.29–1.33	**17.6**	**2.95**	2.84–3.06
High (7–8)	**4.8**	**1.01**	0.98–1.03	**13.7**	**1.07**	1.06–1.09	**23.6**	**1.22**	1.20–1.23	**9.0**	**1.39**	1.34–1.44
Very high (9–10)	**3.9**	1		**13.2**	1		**20.3**	1		**6.4**	1	
No. of cases in model			2,301			2,308			2,311			643
**Age group 2** (25–44 years)	**8.4**			**12.0**			**24.2**			**14.1**		
**Degree of social integration**												
Very low to low (0–4)	**11.5**	**1.29**	1.27–1.32	**15.3**	**1.41**	1.39–1.44	**26.5**	**0.98**	0.96–0.99	**30.7**	**4.29**	4.20–4.38
Medium (5–6)	**12.8**	**1.75**	1.72–1.77	**14.0**	**1.21**	1.20–1.23	**26.7**	0.99	0.99–1.01	**21.8**	**2.88**	2.83–2.94
High (7–8)	**9.2**	**1.31**	1.29–1.32	**12.6**	**1.18**	1.17–1.19	**24.7**	0.99	0.99–1.01	**12.6**	**1.48**	1.45–1.50
Very high (9–10)	**5.9**	1		**10.4**	1		**22.7**	1		**8.9**	1	
No. of cases in model			5,101			5,135			5,144			1,783
**Age group 3** (45–64 years)	**9.7**			**8.2**			**21.7**			**20.2**		
**Degree of social integration**												
Very low to low (0–4)	**20.8**	**2.91**	2.87–2.95	**9.7**	**1.68**	1.65–1.71	**28.8**	**1.53**	1.52–1.55	**42.5**	**4.69**	4.62–4.76
Medium (5–6)	**13.8**	**1.82**	1.80–1.85	**15.0**	**2.70**	2.66–2.74	**26.0**	**1.27**	1.26–1.29	**23.8**	**1.91**	1.88–1.93
High (7–8)	**9.8**	**1.49**	1.47–1.51	**8.1**	**1.48**	1.46–1.50	**21.6**	**1.15**	1.14–1.16	**19.3**	**1.49**	1.47–1.51
Very high (9–10)	**6.1**	1		**5.5**	1		**18.9**	1		**14.0**	1	
No. of cases in model			6,342			6,376			6,395			3,138
**Age group 4** (65+ years)	**16.7**			**5.2**			**10.6**			**23.8**		
**Degree of social integration**												
Very low to low (0–4)	**28.6**	**2.39**	2.35–2.42	**8.4**	**2.55**	2.49–2.61	**12.2**	**1.11**	1.09–1.13	**37.5**	**3.03**	2.98–3.07
Medium (5–6)	**20.3**	**1.66**	1.64–1.68	**6.4**	**1.75**	1.72–1.79	**10.8**	0.99	0.98–1.01	**25.9**	**1.85**	1.83–1.88
High (7–8)	**14.3**	**1.14**	1.13–1.16	**4.5**	**1.14**	1.11–1.16	**9.9**	**0.88**	0.87–0.90	**24.2**	**1.71**	1.69–1.73
Very high (9–10)	**12.1**	1		**3.8**	1		**10.7**	1		**15.9**	1	
No. of cases in model			3,781			3,818			3,849			2,934

Data source: **Swiss Health Survey 2012**

^1)^ Prevalence rates (relative frequencies) according to weighted data

^2)^ Fully adjusted model: OR adjusted for sex, education, foreign nationality and overweight/obesity (BMI >25)

## Discussion

This study aimed to contribute to the existing evidence and research literature on the association between social isolation or loneliness and health, which is largely US-focused, mainly restricted to older people, widely limited to a few single health conditions like depression, cardiovascular disease or all-cause mortality, and mostly based on unidimensional conceptualizations or measures of social isolation [10, 15, 20, 34]. This study therefore addressed these shortcomings and gaps by using nationally representative data for Switzerland and a self-constructed multidimensional index of social integration. In addition, younger age groups were included in the study besides the elderly. Furthermore, various general, mental and physical health conditions were considered including understudied like musculoskeletal disorders or “multimorbidity”. And finally, different health behaviors were taken into account including usually neglected lifestyle factors like the use and consumption of psychotropic medications.

Regarding the first research question, a substantial proportion of almost a quarter of the Swiss resident population aged 15 and older was found to be only partly or rather poorly integrated compared to more than three quarters of the population who were considered and classified as largely or fully integrated. In addition a general and gradual increase in social isolation (lowest degree of integration) with age was observed. Taken together, 17% of the youngsters and adolescents, 20% of the young and middle-aged adults, 23% of those in advanced (working) age and 35% of the elders and pensioners turned out to be only partly integrated or even isolated. Besides the age gradient, a sex difference was observed: women (25%) showed slightly higher proportions of social isolation or partial integration than men (22%). Although great differences in the prevalence of loneliness and social isolation between different countries have been observed and reported for older adults (De Jong Gierveld & Havens 2004), cross-national evidence in this regard is largely lacking. The research literature presents hardly any comparable population-based studies and reports of proportions of socially isolated or only partly integrated people from other countries and across all ages. However, a recent study from England with a nationally representative and weighted sample of the general population aged 16 and above (n = 7,360) found comparable but somewhat lower proportions and sex differences of people who stated that they feel “lonely and isolated from other people” “sometimes” (men: 15%, women: 19%) or “very much” (men: 3%, women: 4%) [39]. In total, about 20% of the English population compared to 24% of the Swiss population as reported in this study seem to be relatively poorly integrated or even isolated. In an earlier prospective cohort study of 16,700 middle-aged French employees, Berkman et al. [29] categorized 26% of 40 to 50-year old men and 35 to 50-year old women as the lowest and second-lowest groups in terms of social integration compared to the 23% of 26 to 45-year old and 21% of 46 to 64-year old workers and non-workers in this study who turned out to be isolated or only partly integrated. This may indicate that France–unlike England–has a somewhat higher proportion of socially isolated people than Switzerland, but this may just as well be attributed to the exclusion of the non-working population or to different assessments and categorizations of socially integrated and isolated people.

With regard to the second research question, the associations between social integration and poor health conditions and behaviors were found to be consistently negative but not equally strong. Particularly strong associations were found for depression and multimorbidity (cumulative health problems), followed by poor self-rated health and use of psychotropic medications. But clear gradients or dose-response relationships were also found for musculoskeletal disorders and for unfavorable health behaviors and lifestyle factors like physical inactivity and unhealthy diet, and less clearly also for regular smoking. The study findings in this regard partly confirm and partly complete the existing literature and evidence. Depressive disorders or symptoms and self-rated health as well as physical inactivity, smoking and even a poor diet have been studied earlier in association with social isolation or loneliness [6, 8–10, 40]. This was occasionally also done in population-based and nationally representative studies [21], but never within an entire population. With regard to accumulated musculoskeletal disorders, multiple health problems and complaints (as a proxy for multimorbidity) and the (mis)use of psychoactive medications, the effects of social isolation have never been studied or reported anywhere so far [10], and particularly not for Switzerland. The findings suggest that social isolation is not only detrimental to general, mental and cardiovascular health but also to musculoskeletal health, and goes along with a substantially elevated risk of using psychotropic medications.

As regards the third research question, significant associations and clear dose-response relationships between the degree of social integration and various health conditions and behaviors were found across all ages, but prevalence rates and gradients sometimes varied strongly between the four studied age groups. Stratified analyses revealed that social isolation is strongly associated with poor health conditions and unhealthy lifestyle behaviors particularly among youngsters and adolescents or among elders and pensioners. Prevalence rates of these health outcomes and behaviors are also highest and/or lowest among one of these two age groups. However and apart from that, consistently finding clear and strong associations and gradients in different (sub)populations–in this case, age groups–is a good indication for causality in the relationships studied, according to Hill’s criteria for causation [41].

### Strengths and limitations

The findings of this study are very distinct and generalizable, i.e. transferable to the general population and other (sub)-populations, since nationally representative and weighted data from the Swiss Health Survey were used. Another strength of the study is the development and use of a multi- or at least two-dimensional measure or index of social integration which promises to be a valid and differentiated indicator of social isolation. Only very few previous studies have considered social isolation as a multidimensional concept [10]. In addition, this study provides evidence for younger ages, particularly for the under-studied age group of 15 to 24 year old youngsters and adolescents, and for a Continental European and German-speaking country like Switzerland for which such evidence is largely or even completely lacking so far. Furthermore, the results of the study include associations between social integration or isolation and musculoskeletal disorders, “multimorbidity” or the use of psychoactive medications, outcomes that have very rarely or never been studied and reported before in this context and in the general population. And finally, the associations studied were controlled and multiple-adjusted for different covariates, and the association analyses were stratified for different age groups. Adjustment and stratification of analyses are successful strategies to test for or exclude confounding. Although based on cross-sectional data, the associations between social integration and health (behaviors) found in this study seem to be valid, generally admitted and not substantially confounded. However, due to the limited design of this study, causal inferences and conclusions cannot be drawn and reversed, and reciprocal causality in the direction of the associations cannot be excluded. People with health problems may therefore be or feel less socially integrated rather than the other way around. There is evidence from longitudinal studies, for example, that depression among adolescents correlates with more loneliness across time [42]. Another point that may be criticized concerns the conceptualization of the main construct. It has been found that social isolation or disconnectedness and feelings of loneliness are not highly correlated with one another [6, 7]. It seems plausible and indeed obvious that one can be socially isolated without feeling lonely and vice-versa. Social isolation and loneliness are consequently regarded as two distinct concepts and social facts by some researchers, whereas in this study they are considered as two aspects or dimensions of the same phenomenon. In any case, being socially isolated *and* feeling lonely–as measured by the social integration index used in this study–is expected to be the tip of the iceberg and a better indication of isolation than just one dimension by itself. In other words, this two-dimensional index and multi-item composite measure presumably is a more valid indicator of social isolation and therefore a stronger predictor or risk factor for poor health than any of its single components. Otherwise associations and gradients probably wouldn’t have turned out to be so strong as misclassification and inconsistency in the measurement usually lead to an underestimation and weakening of the “true” association. However, the validity of this self-constructed two-factorial index could not be tested with an other well-established measure of social isolation or integration since such a measure is not contained in the data set of the Swiss Health Survey.

### Conclusions

The health effects or correlates of social isolation and loneliness have been well-studied for later life, in English-speaking countries like the US or the UK and by using unidimensional measures of social isolation. However, only few and limited evidence is available for younger ages, for continental European and German-speaking countries, and particularly for Switzerland. The same applies to data based on multidimensional measures of isolation. The present study has filled this research gap and has shown that social isolation and disconnectedness are distributed unequally across different age groups in Switzerland but are nevertheless consistently and strongly associated with different poor health outcomes and behaviors in all age groups.

The finding that the proportion of socially isolated or only partly integrated people did not increase progressively with age and that the youngest age group (at 22%) did not show the lowest proportion was rather unexpected. However, it was in line with a previous longitudinal cohort study of 2,232 schoolchildren aged between 5 and 12, born in the mid-nineties in England and Wales, as many as about a quarter of whom were found to be moderately or highly isolated [28]. And although this population-based study consistently found clear dose-response relationships and strong gradients in all associations between social isolation and health (behavior) and for all studied subpopulations (age groups) separately, the associations for most of the considered outcomes (except for unhealthy diet) were even more pronounced in the youngest age group than in the older ones. In other words, the relative risks (odds ratios) were mostly highest and the gradients were steepest among youngsters and adolescents and consequently the results suggest that particular attention should be paid to teenagers and young adults with regard to social isolation and the associated health risks. More research is definitively needed, especially for this age group.

But more than anything and from a public health perspective, the study has clearly identified socially isolated persons–independent of their age and at least for Switzerland–as a relatively small, but important risk and therefore target group for public health initiatives in the future. Measures and efforts against (perceived) social isolation may be a more promising and successful strategy than the usual disease prevention and health promotion activities that are still needed of course.
