# Correction: Health risks associated with social isolation in general and in young, middle and old age

**DOI:** 10.1371/journal.pone.0222124

**Published:** 2019-08-29

**Authors:** Oliver Hämmig

In the Conclusions subsection of the Discussion, there are multiple errors in the second paragraph. The correct paragraph is:

The finding that the proportions of socially isolated and only partly integrated people increase progressively with age and that the youngest age group showed the lowest proportion (17%) and the oldest age group the highest proportion (35%) of socially isolated and only partly integrated was expected and plausible. However, it was not fully in line with a previous longitudinal cohort study of 2,232 schoolchildren aged between 5 and 12, born in the mid-nineties in England and Wales, as many as about a quarter of whom were found to be moderately or highly isolated [28]. And although this population-based study consistently found clear dose-response relationships and strong gradients in all associations between social isolation and health (behavior) and for all studied subpopulations (age groups) separately, the associations for many of the considered outcomes were most pronounced either in the youngest age group or in the oldest one. In other words, the relative risks (odds ratios) were mostly highest and the gradients were usually steepest among youngsters and adolescents or among elders and pensioners. Consequently the results suggest that particular attention should be paid not only to the elderly but equally or even more to the young with regard to social isolation and the associated health risks. More research is definitively needed, especially for the youngest age group.
